# Global change impacts on arid zone ecosystems: Seedling establishment processes are threatened by temperature and water stress

**DOI:** 10.1002/ece3.7638

**Published:** 2021-05-11

**Authors:** Wolfgang Lewandrowski, Jason C. Stevens, Bruce L. Webber, Emma L. Dalziell, Melinda S. Trudgen, Amber M. Bateman, Todd E. Erickson

**Affiliations:** ^1^ Kings Park Science Department of Biodiversity, Conservation and Attractions Kings Park Western Australia Australia; ^2^ School of Biological Sciences The University of Western Australia Crawley Western Australia Australia; ^3^ CSIRO Health and Biosecurity Floreat Western Australia Australia; ^4^ Western Australian Biodiversity Science Institute Perth Western Australia Australia

**Keywords:** arid zone, bet hedging, climate change, drought, germination, grasslands, resilience, restoration, seed dormancy, seed viability, soil moisture, temperature, thresholds, *Triodia*, water availability

## Abstract

Recruitment for many arid‐zone plant species is expected to be impacted by the projected increase in soil temperature and prolonged droughts associated with global climate change. As seed dormancy is considered a strategy to avoid unfavorable conditions, understanding the mechanisms underpinning vulnerability to these factors is critical for plant recruitment in intact communities, as well as for restoration efforts in arid ecosystems. This study determined the effects of temperature and water stress on recruitment processes in six grass species in the genus *Triodia* R.Br. from the Australian arid zone. Experiments in controlled environments were conducted on dormant and less‐dormant seeds at constant temperatures of 25°C, 30°C, 35°C, and 40°C, under well‐watered (Ψ_soil_ = −0.15 MPa) and water‐limited (Ψ_soil_ = −0.35 MPa) conditions. Success at three key recruitment stages—seed germination, emergence, and survival—and final seed viability of ungerminated seeds was assessed. For all species, less‐dormant seeds germinated to higher proportions under all conditions; however, subsequent seedling emergence and survival were higher in the more dormant seed treatment. An increase in temperature (35–40°C) under water‐limited conditions caused 95%–100% recruitment failure, regardless of the dormancy state. Ungerminated seeds maintained viability in dry soil; however, when exposed to warm (30–40°C) and well‐watered conditions, loss of viability was greater from the less‐dormant seeds across all species. This work demonstrates that the transition from seed to established seedling is highly vulnerable to microclimatic constraints and represents a critical filter for plant recruitment in the arid zone. As we demonstrate temperature and water stress‐driven mortality between seeds and established seedlings, understanding how these factors influence recruitment in other arid‐zone species should be a high priority consideration for management actions to mitigate the impacts of global change on ecosystem resilience. The knowledge gained from these outcomes must be actively incorporated into restoration initiatives.

## Graphical abstract

This study demonstrates different response envelopes for seed germination, seedling emergence, and the survival of seedlings and ungerminated seeds after exposure to climate factors associated with climate change. The findings show different thresholds limiting these processes and that seed dormancy is critical for regulating recruitment losses by limiting germination; however, ungerminated seeds are still at significant risk to viability loss. As such, seed bank losses may increase in response to increasing temperature and water stress through climate change.

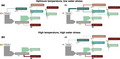

## 1 INTRODUCTION

Arid ecosystems are considered one of the most vulnerable to the destabilizing effects of anthropogenic climate change, as many plant species persisting in these landscapes are already exposed to upper limits of climatic extremes and resource limitations (Allen et al., 2018; Ooi et al., 2009). Coupled with a projected warming trend of 1–5°C in global temperatures in coming decades, the intensity and duration of droughts are expected to increase significantly in arid systems, likely resulting in increased degradation rates and desertification of arid landscapes (Allen et al., 2018; Millennium Ecosystem Assessment, 2005). Without intervention, projected increases in temperature and water stress associated with near term anthropogenic climate change are expected to exceed the physiological limits for many arid zone species. With “nowhere to run,” extreme weather events could lead to widespread extirpation for impacted species that could destabilize entire ecosystems, particularly if keystone taxa are impacted (Parmesan & Hanley, 2015).

Understanding the factors that negatively impact plant recruitment is critical for identifying the management strategies required for intervention (James et al., 2013). The ultimate failure of a species to recruit may occur at one (or several) of the early life‐stage transitions: from a seed to a fully emerged seedling and established plant, through the process of germination, seedling emergence, and survival (Fenner & Thompson, 2005; Grubb, 1977; Larson & Funk, 2016). As seeds transition through these different life stages, their vulnerability to the prevailing environmental conditions also change (Fenner & Thompson, 2005; Hanley et al., 2004; Larson et al., 2020). Germinated seeds that have not yet emerged from the soil profile and recently emerged seedlings are considered to be the most vulnerable of these stages (Larson & Funk, 2016; Saatkamp et al., 2019). For example, previous research has shown that up to 90% of seedling losses occur between germination and emergence (e.g., James et al., 2019; Pyke, 1990). Despite these losses, the mechanisms and sensitivity of emerging seedlings to their surrounding environment are poorly understood.

For the majority of seeds, germination is initially limited by seed dormancy (Baskin & Baskin, 2014). Dormancy is a critical seed functional trait that restricts germination during unpredictable periods in order to direct recruitment to periods that are more favorable for plant establishment (Baskin & Baskin, 2014; Saatkamp et al., 2019). Plant species can maximize their long‐term fitness by dispersing dormant seeds into the soil seedbank (ten Brink et al., 2020). By increasing the variability of seed dormancy in a seed‐set within species and between populations, species bet‐hedge against unfavorable conditions (Baskin & Baskin, 2014; Huang et al., 2016). This variability in seed dormancy also leads to asynchronous germination events over time (Gremer et al., 2016). As seed dormancy can be considered a drought avoidance strategy (Huang et al., 2016; Walck et al., 2011), germination and emergence processes from seeds that do not germinate quickly under favorable conditions tend to be restricted to periods of time when soil moisture availability is high and more sustained (Grubb, 1977; Harper & Benton, 1966). Given the water‐limited nature of arid ecosystems, recruitment flushes from seed populations in the seedbank are relatively infrequent and can lead to low levels of plant regeneration (Mott et al., 1992).

Seeds that do not germinate quickly remain in the soil seed bank and are subsequently exposed to a range of biotic and abiotic filters that influence longer‐term survival (James et al., 2013; Long et al., 2014; Myers & Harms, 2011). Adding complexity to successful plant recruitment are the environmental conditions (temperature and soil moisture) required for successful dormancy alleviation and subsequent germination and emergence (Baskin & Baskin, 2014). Rate of dormancy loss, seed viability decline, and timing of establishment are all at the mercy of these two parameters and vary from species to species (Baskin & Baskin, 2014; Finch‐Savage & Leubner‐Metzger, 2006). For many arid zone species, the thermal tolerances for germination, emergence, and survival of seedlings during early life stages are unknown. Given the increased prevalence of mortality during the seedling emergence phase (James et al., 2011; Larson et al., 2015; Parmesan & Hanley, 2015), understanding the effects of increased temperature and water stress, and identifying the processes that are at highest risk of failure (Larson et al., 2020), will help mitigate future recovery rates for susceptible species and understand recruitment dynamics to climate change.

Understanding the impacts of climate change on recruitment in native ecosystems remains critical. However, such knowledge is equally important for improving the outcomes of revegetation programs, where considerable resources are invested to restore native vegetation across disturbed landscapes (BenDor et al., 2015; Stevens & Dixon, 2017). The direct seeding of native species is currently the most cost‐effective method of returning plants to degraded ecosystems (Merritt & Dixon, 2011), but efforts routinely fail to meet restoration targets due to poor plant establishment (James et al., 2019; Kildisheva et al., 2016). It is important to understand the mechanisms driving survival and mortality during early stages of recruitment to improve return on investment when restoring degraded ecosystems. Moreover, despite its ecological benefit, seed dormancy still poses a major challenge for achieving successful restoration outcomes with certain species (Kildisheva et al., 2019; Lewandrowski et al., 2017; Wagner et al., 2011). In the context of arid‐zone restoration, where some species may inhabit areas where climatic conditions are near to their physiological thresholds, it is crucial to understand which of the processes between seed and established seedling are most vulnerable to the increasingly hostile conditions associated with climate change.

The genus *Triodia* R. Br. are keystone grass species of Australia's arid interior, covering ca. 1.8 M km^2^ (Griffin, 1990), and is a major framework group of species used in Australian arid zone restoration (Erickson et al., 2017). Despite their dominance in natural ecosystems, reinstating *Triodia* grasslands is one of the most significant challenges for restoration practitioners (Smyth et al., 2012). Low recruitment rates from dispersed or broadcast seeds have been associated with a high prevalence of seed dormancy (Shackelford et al., 2018; Wright & Fensham, 2017) and reliance on sustained water availability to support germination and subsequent seedling emergence (Lewandrowski et al., 2017). Despite our ability to manipulate dormancy to increase the germination response of seeds under laboratory conditions (Erickson et al., 2016; Lewandrowski et al., 2018), these treatments have not translated to a comprehensive increase in the performance of seeds that are broadcasted during field trials. Coupled with the projected increases in soil temperatures and water stress associated with climate change in the Australian arid zone (Department of Primary Industries & Regional Development, 2020), future restoration of *Triodia* species in Australia is likely to become even more difficult. Understanding the constraints to the seed recruitment potential of *Triodia* through the critical initial stages of regeneration (i.e., germination, emergence, and survival) may allow us to pinpoint the life stages most vulnerable to future climate change and improve restoration outcomes.

In this study, we used controlled environmental chambers to assess the combined effects of seed dormancy, increased temperature, and water stress in six *Triodia* species to determine which demographic process is at highest risk of failure under a range of simulated future climate scenarios. Given that alleviation of seed dormancy widens the environmental envelope under which germination can occur (Bradford, 2002; Lewandrowski et al., 2018), we anticipate that the stages of seedling emergence and survival will be most at risk from increased temperatures and water stress. We also expect that the viability of ungerminated, less‐dormant seeds may be more susceptible to decline under increased temperatures and water stress compared with more dormant seeds. Further, we expect the effects of seed dormancy, temperature, and water stress to vary between species in this study (e.g., Baskin & Baskin, 2014; Huang et al., 2016). We aim to provide a process‐based insight into the effects of elevated temperature under contrasting moisture availability on the recruitment potential in *Triodia*, including the potential fitness trade‐off involved in alleviating dormancy prior to seed broadcasting.

## 2 MATERIALS AND METHODS

### 2.1 Study region, species selection, and seed processing for experiments and initial seed viability

All seeds used in our experiments were sourced from the Pilbara region of Western Australia. The Pilbara is one of the largest mining regions in Australia, with over 230,000 ha of disturbed landscapes requiring active restoration to reinstate native vegetation (Erickson et al., 2017). The Pilbara is characterized by warm (10–25°C) winters and hot (25–45°C) summers, with annual rainfall ranging between 300 and 350 mm, with approximately 70% occurring over summer (December–March) (Charles et al., 2013).

Seeds from six *Triodia* species (*T*. *basedowii* E. Pritz, *T. epactia* S.W.L. Jacobs, *T. lanigera* Domin, *T. pungens* R. Br., *T*. *vanleeuwenii* B.M. Anderson & M.D. Barrett, and *T. wiseana* C.A. Gardner) were collected by commercial suppliers in March 2011 (Nindethana Seed Service, and Native Bushland Seed Supply) and supplied to Kings Park and Botanic Garden (Kings Park Science, Department of Biodiversity, Conservation and Attractions). Seeds were stored at King Park in a cool dry storage facility, where relative humidity (15% RH) and temperature (15°C) were maintained at constant levels prior to experimental use in 2014. Seed dormancy and viability were maintained under these storage conditions, determined via frequent seed testing that occurred prior to experimentation, and followed current restoration seed storage protocols for this species as described by Erickson and Merritt (2016). Floret fill in *Triodia* is characteristically low and variable (Erickson et al., 2016), which was managed by concentrating filled florets using a vacuum aspirator (“Zig Zag” Selecta, Machinefabriek BV, Enkhuizen, the Netherlands) that removed loose plant material and empty floret husks from florets that contained viable seeds (i.e., “filled”).

Seed dormancy in *Triodia* is multi‐layered, with the physiologically dormant seed encapsulated by floral bracts that restrict embryo growth (Erickson, Shackelford, et al., 2016). By removing the floret, dormancy can be alleviated to a degree and seeds are generally found to germinate under a wider environmental envelope for temperature and water stress when compared to intact florets (Erickson, Shackelford, et al., 2016; Lewandrowski et al., 2018). Given this response, we tested two dormancy states: (i) dormant, intact florets; and (ii) less‐dormant, cleaned seeds (Lewandrowski et al., 2017). The intact florets were left untreated while the cleaned seed treatment was achieved by careful separation of florets and seeds through a 1.4 mm sieve that removed the ancillary bracts. These seeds were re‐aspirated and checked under a dissecting microscope and X‐ray (Faxitron MX‐20 X‐ray cabinet, Tucson, Arizona, USA) to select only undamaged seeds (i.e., with a fully intact embryo and no visible damage to the endosperm) for use in the trials. A viability test was conducted on the dormant intact florets and less‐dormant, cleaned seeds using four replicates containing 25 intact florets and cleaned seeds by incubating on 0.7% H_2_O‐agar at 25°C, 30°C, 35°C, and 40°C, testing the warmer ranges for temperature (see Figure S1). At the completion of the viability test, ungerminated seeds were cut‐tested to inspect seed viability, whereby a firm, white endosperm and embryo in the seed indicated healthy living tissue and was considered viable.

### 2.2 Effects of temperature and water stress on germination, emergence, and survival from intact florets and cleaned seeds

#### 2.2.1 Soil characteristics

Locally sourced topsoil—an iron‐rich sandy clay loam—was obtained from mining operations in the southern Pilbara region, Western Australia. Prior to experimental use, soil was sieved to <2 mm particle size to remove large gravel fragments and to reduce variation in soil water potential treatments during the experiment. The sieved soil was then dried in an oven (60°C for 72 hr) and homogenized before storage. Soil texture analysis was conducted using a Mastersizer (Malvern Instruments, Worcs., United Kingdom) to determine particle size following the USDA particle size classification and resulted in fractions of 82%, 14%, 4% sand, silt, and clay content, respectively. Soil water retention curves were determined using a dewpoint psychrometer (WP4C Dew Point PotentiaMeter, Decagon Devices, Inc.) where soil samples were saturated and periodically dried at 75°C and then measured to obtain the soil water potential. The water retention curves were used to calculate water potentials for experimental thresholds from gravimetric soil water weights using the van Genuchten model (1980).

#### 2.2.2 Experimental trial conditions in controlled climate chambers

**FIGURE 1:**
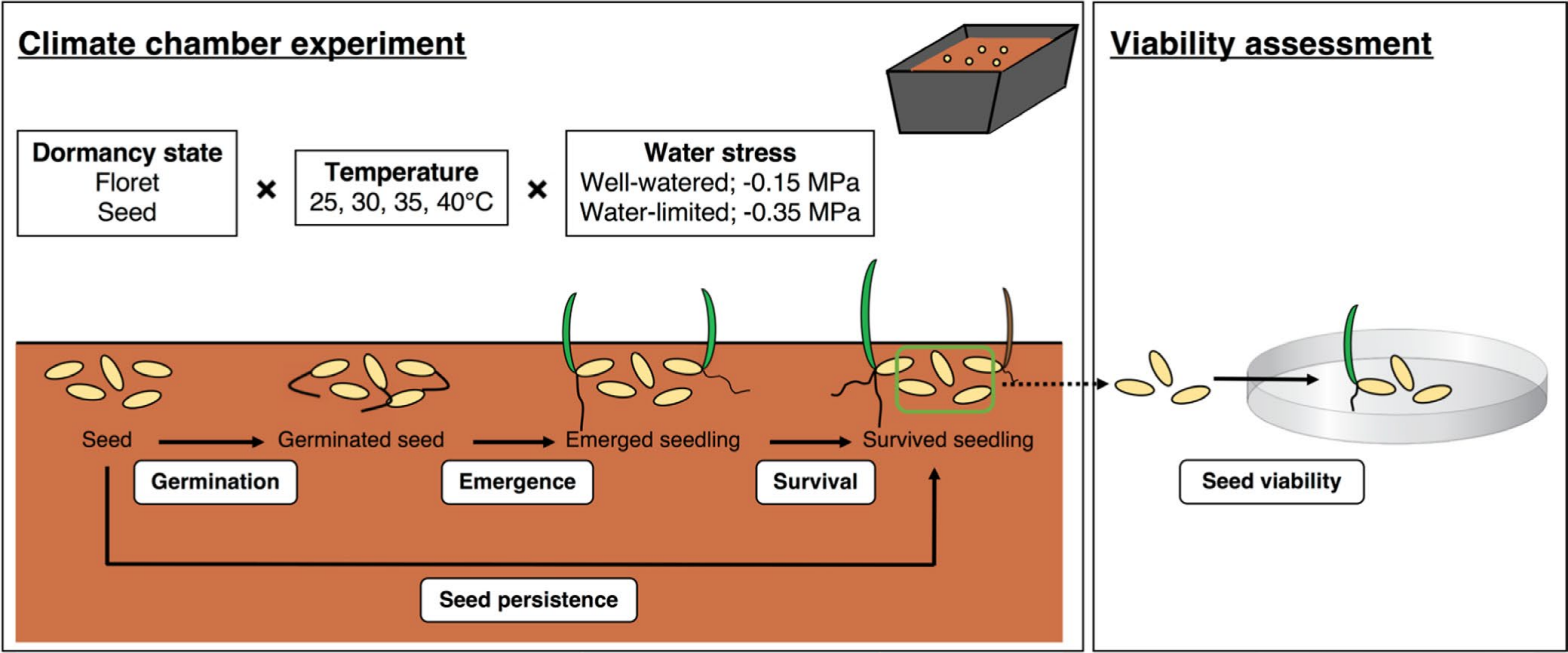
Climate chamber experiment testing dormancy state, temperature and water stress on germination, seedling emergence and survival; and petri dish experiment at the conclusion of the climate chamber study testing seed persistence by assessing viability. The climate chamber was conducted over a 30‐day period, whereby germination was assessed at the completion of the trial by separating florets and seeds from the soil and counting germinated from ungerminated proportions, while seedling emergence and survival scored every 2 day during the trial period. The ungerminated seeds (dashed arrow) were then assessed for viability in the petri dish experiment

A seedling emergence trial was conducted on floret and seed material from the six *Triodia* species (from December 2013 to February 2014) using multiple controlled condition growth cabinets (40 μmol PAR, 12 hr alternating light/dark cycle (50% RH) (Shin Saeng Scientific Co. Ltd., Gyeonggi‐do, South Korea). Seeds of *Triodia* species have wide temperature envelopes for germination, with maximum responses observed between 15 and 40°C (Lewandrowski et al., 2018). Four constant ambient temperatures (25°C, 30°C, 35°C, and 40°C) were used to assess the influence of temperature on seedling emergence (Figure 1). Prior to seeding, seedling punnets (0.38 L; 45 mm × 12 mm × 70 mm) were filled with oven‐dried topsoil to a uniform weight. Each punnet with topsoil was then hydrated with reverse‐osmosis water, close to field capacity (−0.01 MPa) and allowed to air dry to the desired moisture threshold equivalent to soil water potential thresholds (Ψ_soil_) of either −0.15 MPa (well‐watered, control conditions), or −0.35 MPa (water‐limited conditions) (Figure 1). Per species, 13,760 seeds were sown, whereby each temperature (25°C, 30°C, 35°C, and 40°C) × water stress treatment (well‐watered, control; and water‐limited condition) consisted of four replicate punnets sown with 215 intact florets or cleaned seeds and covered with 5 mm of topsoil. To more accurately maintain the experimental moisture thresholds in treatments, a thin 5–7 mm layer of transparent, low density (0.92 g/cm^3^) alkathene beads (Qenos Pty Ltd, Australia) was applied over the soil surface to reduce evaporative moisture loss. The final experimental punnet weights were recorded in order to monitor and maintain experimental water‐availability thresholds. Re‐hydration to target Ψ_soil_ was conducted every 2 days, and Ψ_soil_ monitored gravimetrically daily over the trial period to ensure consistent treatment hydration. Periodically, unseeded punnets replicating the same emergence conditions of either −0.15 MPa or −0.35 MPa were destructively harvested and soil samples tested with a dewpoint psychrometer (WP4C Dew Point PotentiaMeter, Decagon Devices, Inc.) to monitor Ψ_soil_‐thresholds. Water loss from the punnets never exceeded 5 g of water between hydration periods, which equated to approximately Ψ_soil_ of −0.01 MPa in the −0.15 MPa treatment and −0.05 MPa in the −0.35 MPa treatment.

#### 2.2.3 Demographic transitions between seeds and seedlings: germination, emergence, and survival in the climate chamber study

Demographic life stages and transitions were defined following Fenner and Thompson (2005), whereby *germinated seed*, *emerged seedling*, and *survived seedling* are the assessed life stages, and *germination*, *emergence*, and *survival* the transitions between the respective life stages. As the study was concerned with understanding the implications of dormancy on recruitment processes, we followed the 30‐day rule for the climate chamber experiment as described in the dormancy classification system of Baskin and Baskin (2014). Germination was assessed at the end of the emergence trial period, by carefully sifting the soil through fine‐meshed sieves (1–1.14 mm mesh‐grades). The exhumed florets or seeds were inspected under a dissecting microscope and germination confirmed when radicle protrusion was observed to be one third of the floret/seed length. During the 30‐day period, seedling emergence and survival scored every 2 days. Seedling emergence was defined by the total number of seedlings that emerged with the protrusion of fully expanded cotyledon through the soil and bead surface over the 30‐day trial period. Emergence speed is summarized by the time to 50% emergence parameter in Table S4. Seedling survival was quantified by subtracting the number of dead seedlings, whereby a seedling was considered dead upon tissue desiccation following wilting of the cotyledon/ and the primary leaf when present, from the total number of seedlings that had emerged. The majority of seedling mortality occurred within the first 4 ​days following emergence—a consistent trend across all species in the climate chamber experiment.

#### 2.2.4 Viability assessment on ungerminated intact florets and cleaned seeds

An experiment was conducted to specifically assess the viability of seeds/florets after the climate chamber study (Figure 1). Ungerminated intact florets, and cleaned seeds that were removed from the soil punnets when assessing for germination previously, were transferred into Petri dishes containing 0.7% H_2_O‐agar to determine the germinability of seeds. Four replicates of 75 florets or cleaned seeds were randomly selected from the pool of remaining intact florets/seeds from each temperature × water stress treatment under optimal conditions (30°C, 12 hr alternating light/dark) for 30 days. At the completion of this experiment, germination was counted and ungerminated florets/seeds subjected to a cut‐test. From the cut‐test, dead and alive proportions were assessed, whereby live seeds displayed a firm, white endosperm and embryo, indicated healthy living tissue. Viability was determined as the total number of seeds that germinated summed with the total number of ungerminated seeds found to be alive during cut testing.

### 2.3 Statistical analysis

All analyses were conducted in the statistical environment R (R Core Team, 2018) using the “lme4” (Bates, 2010), “car” (Fox, 2015; Fox et al., 2007), and “sjPlot” (Lüdecke, 2019) packages. Germination, emergence, and seedling survival responses from the climate chamber experiment, and the germination and viability assessment were analyzed using generalized linear mixed‐effects models (GLMMs) that were fitted with a logit‐link function and a binomial error structure. To account for random, uncontrolled effects resulting from the soil substrate and the climate chamber during the trial, for each species each replicate punnet was nested within every experimental treatment and classified as random effect for each species. In each GLMM, the transitions (a binary assessment: success or failure) between *germinated seed*, *emerged seedling*, and *survived seedling* were dependent on the prior success numbers for each life stage. That is, “germination” responses were dependent on the prior total number of sown seeds; “emergence” responses were dependent on the prior total number of germinated seeds; and “survival” responses were dependent on the prior total number of emerged seedlings after 30 days. In order to determine predictive links between transitions and factors, a multifactorial GLMM was constructed using species, dormancy state, temperature, and water stress as categorical factors for each transition. The experimental design was initially fully crossed for each species × dormancy state × temperature × water stress treatment across replicates (*n* = 384 observations). However, emergence (*n* = 282 observations) and survival responses (*n* = 229 observations) changed across the experimental design due to NA responses in replicate punnets for emergence following germination responses that were zero, and similarly for survival responses, when emergence responses were zero (see available data source in the Dryad repository). The NA responses resulted in a decreased number of observations in treatment combinations, and in some cases caused issues with model fitting when accounting for interactions. As such, we dropped interactions in the GLMM models. In order to make consistent comparisons between germination, emergence, and survival responses, the model structure for the fixed effects was species + dormancy state + temperature + water stress. Given our hypothesis that following seed dormancy alleviation, seedling emergence, and survival stages would be the most at risk from increased temperature and water stress, the reference for the regression was determined as the dormant floret for dormancy state, under well‐water conditions for water stress, at 25°C for temperature, while the intercept for all species was removed. Forest plots for the main effects were then used to visualize the effect size of the factors using log‐odds ratio coefficients for species dormancy state, water stress, and temperature. The log‐odds ratio coefficients for germination, emergence, and survival events from the main effects were then converted into probability ratios using the inverse logit function. Further, to determine model fit and quality of the models, we calculated the marginal *R* ^2^‐values (*R* ^2^_m_), which considers the variance of the fixed effects, and conditional *R* ^2^‐values (*R* ^2^_c_), which considers the variance of both fixed and random effects in the model as described by Nakagawa and Schielzeth (2013). GLMMs were further employed to assess germination viability of seeds from within intact florets or cleaned seeds after the climate chamber study, following a similar structure as constructed in the climate chamber experiment. Complete tables containing summary statistics are available in the supplementary materials (Table S2.1 and S2.2).

In order to assess the overall impact of increased temperature and water stress across our six study species, individual species responses were averaged across the different life‐stage transitions using the “summarySE” function in the “Rmisc*”* package (Hope, 2013). Sankey diagrams are then used to depict the fate of seeds and florets under optimal (25°C, nonlimited water) and future climate (40°C, water limited) scenarios.

## 3 RESULTS

### 3.1 Effects of dormancy state, temperature, and water stress on germination, seedling emergence, and survival

**FIGURE 2:**
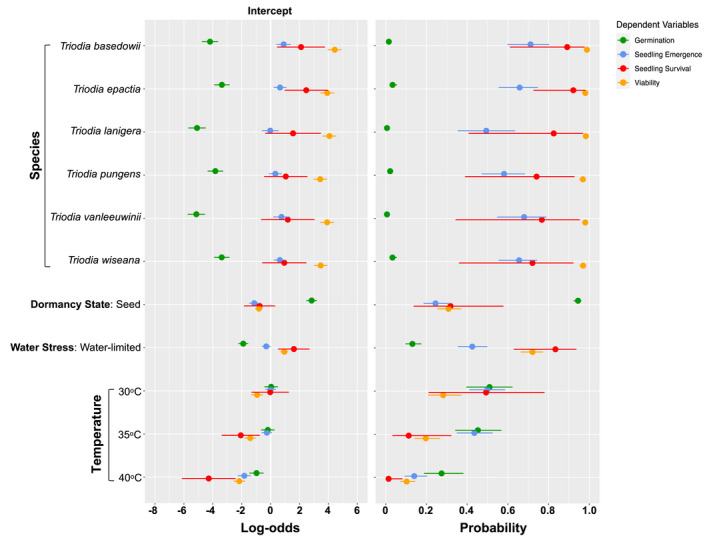
Summary forest plot from GLMMs showing the log‐odd ratios and converted probability ratio estimates (±95% confidence intervals) for germination (green), emergence (blue), survival (red), and viability (orange) responses for six *Triodia* species, dormancy state, water stress, and temperature. The intercept for the regression was determined as the dormant floret, under well‐watered conditions, at 25°C, while the reference for species was removed

**FIGURE 3:**
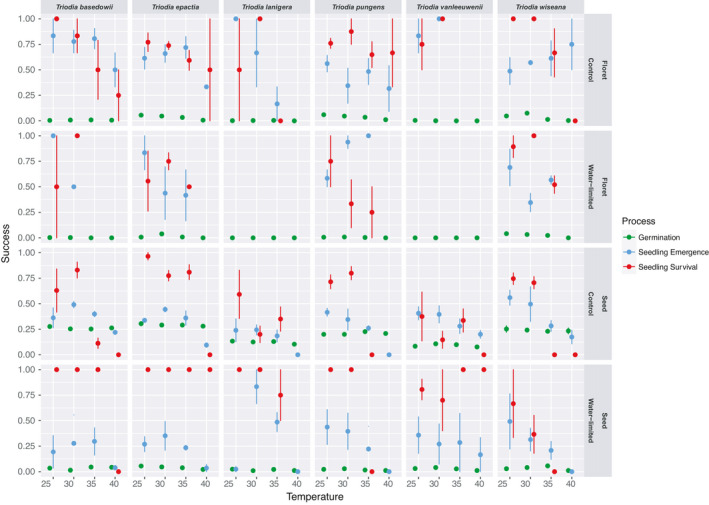
Germination, emergence, and survival success from intact florets and cleaned seeds from six *Triodia* species. Dormant intact florets, and less‐dormant, cleaned seeds (both *n* = 215, per punnet (*n*_rep_ = 4) were sown into replicate punnets containing topsoil and incubated in a climate chamber at well‐watered (control) and water‐limited conditions incubated at constant 25°C, 30°C, 35°C, and 40°C temperatures. The error bars indicate standard errors of the mean. Ratios are presented as transition success proportion from the preceding life stage

The overall models describing the effects of species, dormancy state, temperature, and water stress showed each of these factors differed in shaping the response for germination (*R* ^2^_m_ = 0.484; *R* ^2^_c_ = 0.558), emergence (*R* ^2^_m_ = 0.204; *R* ^2^_c_ = 0.208), and survival (*R* ^2^_m_ = 0.334; *R* ^2^_c_ = 0.631) (Figures 2 and 3).

Germination was the life‐stage transition most affected by dormancy state, as demonstrated by the low germination capacity of florets (0%–8%, Figure 3) and the significant germination increase for cleaned seeds (log‐odds ratio = 2.84, probability ratio = 0.94; z = 15.49, *p* < .001, Figure 2; 10%–35%, Figure 3). After dormancy state, water stress (log‐odds ratio = −1.89, probability ratio = 0.13, z = −10.89, *p < *.001) and a temperature of 40°C significantly reduced germination (log‐odds ratio = −0.97; probability ratio = 0.27, z = −3.89, *p < *.001), but the effect size for temperature was comparatively smaller in contrast to dormancy state and water stress (Figure 2).

Overall, less than 15% of the total number of sown florets and cleaned seeds reached the emerged seedling stage and less than 12% of emerged seedlings survived to 30 d (Figure S3.1 and S3.2). The proportion of seedlings emerging was more variable (0%–100%) in comparison to the germination response (0%–35%). Temperature more strongly influenced emergence and survival compared with germination (Figures 2, 3 and 2, 3). Specifically, high temperatures of 40°C were associated with a greater reduction in seedling emergence than germination (log‐odds ratio = −1.82, probability ratio = 0.14, z = −7.90; *p < *.001, Figure 2), and 35°C conditions significantly reduced survival after emergence (log‐odds ratio = −2.06; probability ratio = 0.11, z = −3.06, *p < *.001). Water‐limited conditions only marginally reduced seedling emergence (log‐odds ratio = −0.3, probability ratio = 0.43, z = −1.98, *p = *.048), and the capacity to survive after emergence was relatively high (log‐odds ratio = 1.61, probability ratio = 0.83, z = 2.91, *p < *.004). Seedlings generated from cleaned seeds had significantly lower seedling emergence (log‐odds = −1.13, event probability = 0.24, z = −6.36, *p < *.001) and overall similar survival (log‐odds = −0.76, event probability = 0.32, z = −1.39, *p* = .166) than seedlings generated from intact florets (Figure 2). While germination still occurred at 40°C from cleaned seeds under well‐watered conditions, emergence varied between 0% and 25% (Figure 3). From the cleaned seeds that did emerge at 40°C, there were no surviving seedlings after 30 d (Figure 3). In contrast, while germination at 40°C under well‐watered conditions was low from intact florets, seedling emergence from germinated florets varied between 31% and 75%, and survival following emergence varied between 0% and 50% (Figure 3).

On a species level, responses varied more strongly for germination in contrast to emergence and survival (Figure 2). Higher germination was generally associated with *T. epactia* (log‐odds ratio = −3.82, probability ratio = 0.03, z = −12.38) and *T. wiseana* (log‐odds ratio = −3.38, probability ratio = 0.03, z = −12.47, both *p < *.01), while species that were the most restricted in their capacity to germinate were *T*. *lanigera* (log‐odds ratio = −5.09, probability ratio = 0.01, z = −16.71) and *T*. *vanleeuwenii* (log‐odds ratio = −5.14, probability ratio = 0.01, z = −16.72, both *p < *.01). Higher emergence and survival were generally associated with *T. basedowii* (Figures 2, 3 and 2, 3), while *T. pungens* and *T. wiseana* demonstrated the lowest proportions compared with the other species (Figures 2, 3 and 2, 3).

Across all species and under optimal conditions (well‐watered conditions at 25°C, Figure 5) for recruitment, seed germination was significantly higher for the less‐dormant seeds compared with the more dormant florets (3% and 21%, respectively; Figure 5). However, for the 3% of florets that successfully germinated, 67% of these seeds emerged and 82% survived, compared with 39% successful emergence and 33% survival from seeds. Comparatively, there was no germination, emergence, or survival of florets across the six species when incubated under high temperature and water stress. For seeds, germination (2%) and emergence (4%) were low.

### 3.2 Effects of temperature and water stress on ungerminated seeds

**FIGURE 4:**
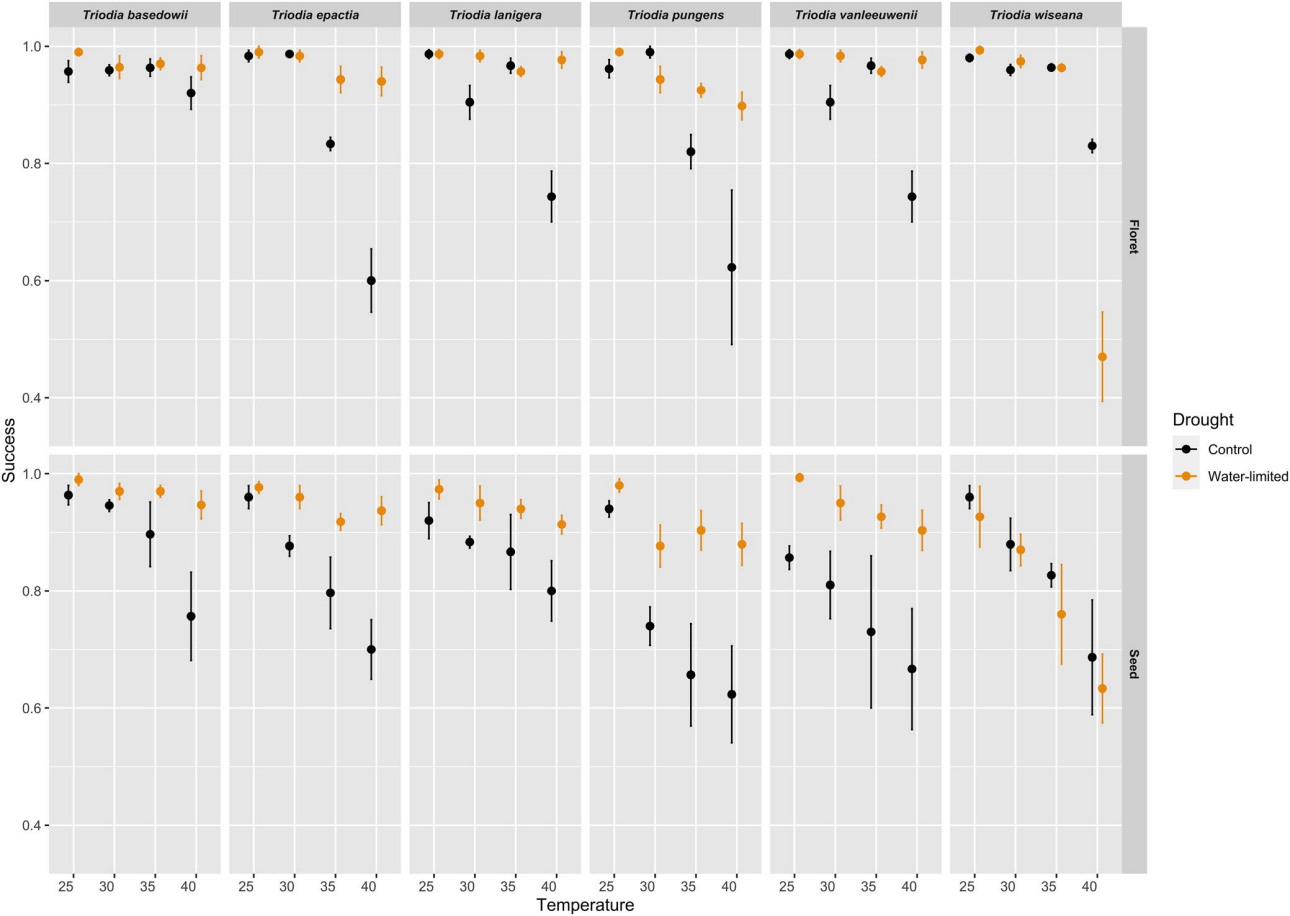
Post climate‐chamber viability of intact florets (black) and cleaned seeds (orange) (*n* = 75 per replicate) after incubating for 30 days under conditions considered optimal (30°C; 12 hr alternating light/dark conditions) to determine dead and live proportions. Viability is defined as the number of germinated and ungerminated, viable intact florets, or cleaned seeds. Error bars indicate standard errors of the mean, *n* = 4

The viability of ungerminated seeds and seeds retained within florets varied significantly across dormancy state, temperature, and water stress at the completion of the 30‐day climate chamber experiment (Figure 2). On average, viability was consistently highest for both intact florets and cleaned seeds in the water‐limited treatment at 25°C (92%–99%; Figure 4). Viability was negatively affected by temperature (log‐odds ratio = −2.17, probability ratio = 0.16, z = −10.78, *p < *.001), under well‐watered conditions (Figures 2, 4 and 2, 4, log‐odds ratio = 0.95, probability ratio = 0.58, z = 6.84, *p < *.001). Viability also decreased significantly under these conditions in the intact florets in comparison with cleaned seeds (log‐odds ratio = −0.81, probability ratio = 0.33, z = −5.82, *p < *.001).

On a species level, the lowest viability was observed in *T. wiseana* (log‐odds ratio = 3.47, probability ratio = 0.97, z = 14.02, *p < *.001) and *T. pungens* (log‐odds ratio = 3.44, probability ratio = 0.97, z = 14.59, *p < *.001) (Figures 2, 4 and 2, 4), followed by *T*. *vanleeuwenii* (log‐odds ratio = 3.91, probability ratio = 0.98, z = 16.32, *p < *.001), while *T. epactia* and *T*. *basedowii* maintained highest viability (Figures 2, 4 and 2, 4).

**FIGURE 5:**
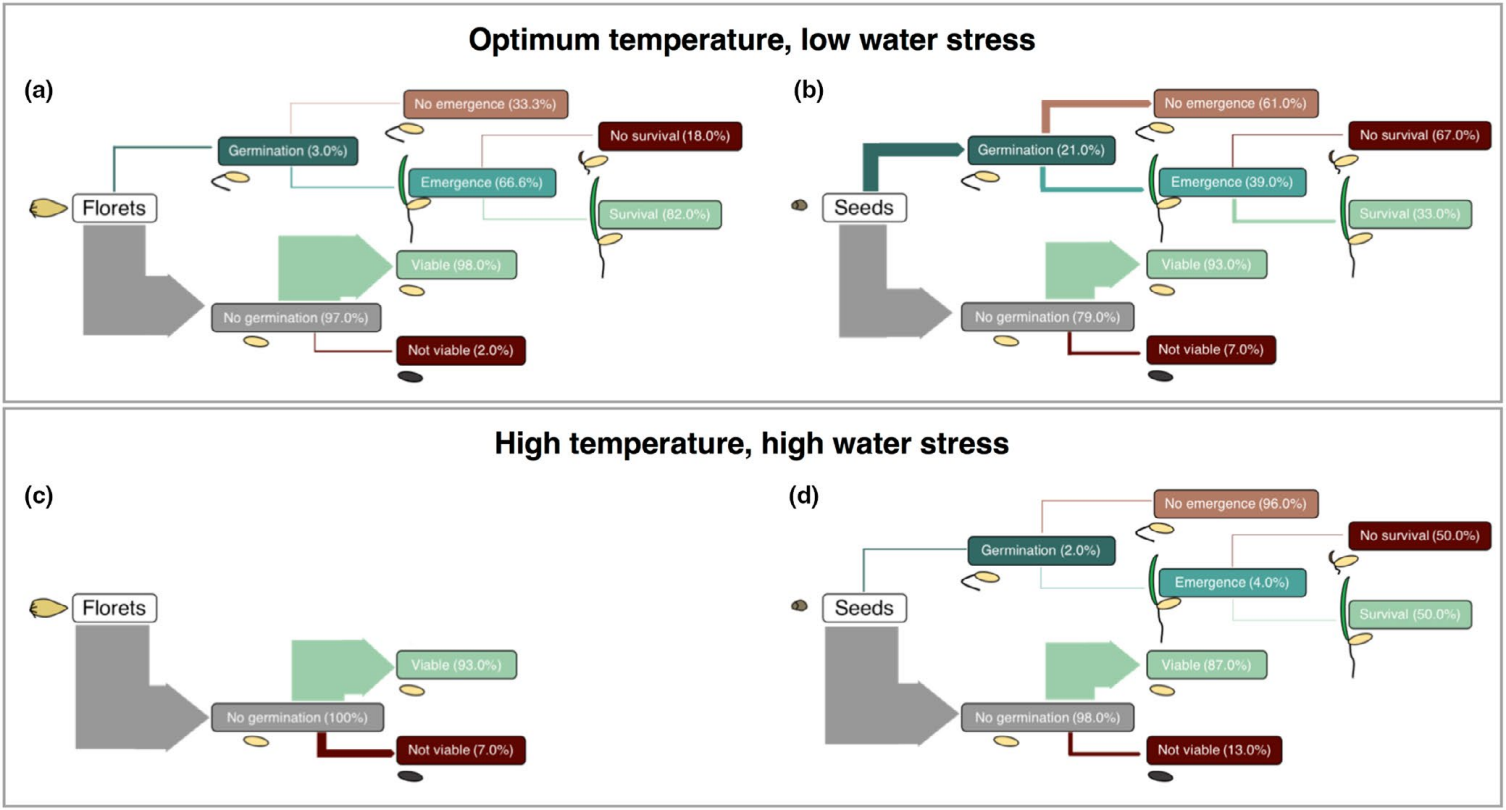
Sankey diagram summarizing the recruitment life‐stage transitions across six keystone *Triodia* species under optimal (*a, b*; 25°C and low water stress) and potential future climatic conditions (*c*, *d*; 40°C and high water stress). The width of each transition arrow represents the total proportion of the initial seed lot (i.e., 100%) that transitioned to each of the life stages, while the percentages are the number of seeds that transitioned to that life stage as a percentage of the previous life stage

Across all species and under optimal conditions for recruitment, the viability of florets was maintained at 98% and for seeds 95% (Figure 5). Under high temperature and water stress, floret viability remained high (93%), but was reduced to 87% for seeds.

## 4 DISCUSSION

A failure along the way from seed to established seedling can arise when the environmental conditions that influence the germination process are not favorable and do not enable subsequent seedling emergence, establishment, or survival (Grubb, 1977; Harper, 1977). Our results demonstrate that dormancy state, temperature, and water stress have different impacts on germination, emergence, and seedling survival processes during recruitment of the arid‐zone grass genus *Triodia*. Dormancy state was the most critical factor regulating germination, while temperature and water stress were more influential during seedling emergence and survival. Unsurprisingly, there were higher germination proportions from the less‐dormant seeds under the experimental conditions. However, under increased temperatures (e.g., 35–40°C relative to 25°C), a higher proportion of the less‐dormant seeds transitioned between the germination and emergence phases but did not survive, contributing to an overall higher loss of viable seeds from the seed bank. While the persistence of seeds in both dormancy states was compromised under warm and wet conditions (Figure 4), the viability from the more dormant intact florets was higher compared with the less‐dormant, cleaned seeds. Taken together, these results indicate that the more dormant florets provide a protective mechanism to reduce mortality before and after germination in these six *Triodia* species. Considering these results, the dormancy state in grass florets is a critical factor in regulating both seedbank and recruitment dynamics and stability, which has important implications for the future of natural and degraded grassland ecosystems.

Our study demonstrates that mortality is highest during the emergence phase, like many other arid‐zone species (James et al., 2011; Larson et al., 2015; Muñoz‐Rojas et al., 2016a, 2016b), but also supports the importance of seed dormancy as a critical functional trait that assists in minimizing losses before and after committing to germination (Baskin & Baskin, 2014; Fenner & Thompson, 2005). Emergent seedlings resulting from the more dormant florets demonstrated higher survival proportions than germinated and emerged seedlings. For instance, the final number of surviving seedlings of *Triodia pungens* at 35°C under well‐watered conditions was two seedlings (=25%, *n* = 8 germinated seeds) within florets, whereas the equivalent from cleaned seeds was zero surviving seedlings (*n* = 49 germinated seeds). There were similar trends observed from other species, for example, *T. wiseana* with zero surviving seedlings from 50 germinated seeds, and two surviving (=28.5%) seedlings from seven germinated florets under well‐watered conditions. These trends become more evident when generalizing across species (Figure 5), whereby seedlings generated from the more dormant florets demonstrate 82% survival after emergence, compared with 33% when seedlings emerged from the less‐dormant seeds under optimal temperature and low water stress conditions. While it is well established that the floret imposes several blocks to germination of the seed within its covering structures (i.e., palea and lemma; sensu Adkins et al., 2002; Bewley et al., 2013), recent research has hypothesized that an increase in vigor of *Triticum turgidum* var. *dicoccoides* seedlings may be related to a range of beneficial chemicals (e.g., nutrients, hydrolases with antifungal properties, reactive oxygen species‐detoxifying enzymes etc.) contained within the maternal floret structure (Raviv et al., 2017). Although germination was very low amongst florets in our study, the seedlings that did emerge may have benefited from similar substances within the floret, which may have aided establishment.

While our results show that a short‐term increase in germination may be gained by cleaning seeds from their covering structures, there is a clear trade‐off between short‐term gains in germination and long‐term survival and persistence. For instance, when seed dormancy was overcome by cleaning the seed, the risk avoidance strategy inherent in the floret was removed, allowing germination to proceed at higher temperatures, but the emerged seedlings were generally less likely to survive (Figure 5). In this regard, seeds that remained in the protective floret structure were able to avoid germination and mortality events under unfavorable conditions (e.g., Lewandrowski et al., 2018). As a result, the recruitment numbers remain low, but the establishment risk is reduced, cued to milder conditions in the germination niche and likely spread over time (Barga et al., 2017). Therefore, total germination and survival under field conditions over time may actually be increased for seeds that remain in the protective floret, ultimately leading to more successful restoration outcomes.

The findings of this study have important implications at multiple levels for ecosystem resilience to global climate change, as well as for the management responses we consider for improving restoration and conservation outcomes. First, and specifically for *Triodia* community dynamics, the findings reveal significant variation between species in their germination niche and, therefore, how projected climate change will impact their fitness. While all species were capable of germinating across the experimental temperatures, there was variation among species between the responses of intact florets and cleaned seeds, which may be explained by varying dormancy levels previously reported for these species (e.g., Erickson, Shackelford, et al., 2016; Lewandrowski et al., 2018). The species were also found to vary in response to high temperature at the different recruitment stages. For instance, *T. wiseana* demonstrated highest viability decline and highest seedling mortality, while species that were low in their germination capacity (*e.g*.*, T. basedowii,* *T. lanigera,* and *T. vanleeuwenii*) were generally associated with lower viability decline and increased seedling survival. For *T. basedowii*, *T. lanigera*, and *T. vanleeuwenii*, germination was generally the lowest among all species, which may suggest that these species are more strongly bet hedging compared with the other species we tested. The greater viability maintenance of seeds and increased seedling survival after emergence may also be more favorable traits in arid landscapes and possibly under changing climate regimes. In contrast, the species such as *T. epactia*, *T. pungens*, and *T*. *wiseana* are generally associated with more mesic ecosystems compared with the other species (Lazarides, 1997), which may explain their increased sensitivity to high temperature and water stress.

It is difficult to predict how these species will perform in the long‐term, as such insight requires further field testing to quantify seed persistence–community composition interactions at increasing time scales and under changing climate scenarios (Long et al., 2014; Parmesan & Hanley, 2015). Based on these findings, it is likely that the species may change in their abundance on a local scale in the short‐term, with species capable of preventing high net losses of seeds out of the seed bank through viability decline or seedling mortality likely becoming more abundant as the landscape becomes hotter and drier. To date, there are no published investigations into these finer‐scale recruitment dynamics and their implications on changes in dominance in *Triodia* and across perennial species in the arid zone in general, highlighting a major gap in our scientific knowledge. Despite displaying a wide environmental envelope for germination, and fast emergence speed of the *Triodia* species (time to 50% emergence, 3–7 days at 30°C; see Supplementary Information, Table S4), germinated seeds have a limited window of opportunity to grow rapidly before temperatures exceed tolerance thresholds, and thus risk the success of recruitment. Taking into account the species‐level differences in thermal and water stress tolerances, it is possible that these species may change in their local abundance in natural systems, highlighting the importance of closely monitoring changes in grassland composition as climate‐associated changes unfold.

Second, from a broader perspective of the ecology of arid grasslands, the results suggest that we could expect more infrequent recruitment events due to elevated soil temperatures. Current predictions in the Pilbara, northwest Australia, suggest substantial temperature increases within this century (by 2030: 0.6–1.5°C; by 2090: 1.5–3.1°C; Department of Primary Industries & Regional Development, 2020), which will push the tolerance envelope for many species in this region and compromise plant regeneration from seeds. This recruitment risk has previously been noted for other arid zone areas across Australia (Cochrane, 2020; Ooi et al., 2009) and globally (James et al., 2019; Renwick et al., 2018). A possible outcome of this trajectory could be range contraction for many species, should they fail to recruit under unfavorable microclimatic conditions in the niche or adapt and survive under projected warming and drying conditions. As such, a greater understanding of the adaptive capacity of seeds, seedlings, and adult plants is needed in order to reduce our uncertainty about the range of possible future responses and range distribution of arid zone species in general. Arid ecosystems more broadly could change significantly in their structure and composition should keystone species fail to recruit under increasing temperature and water stress conditions that are exacerbated by climate change. To better understand and predict these changes, future studies should consider measuring recruitment rates of arid zone species under manipulated climate scenarios in the field.

Last, as this study only quantifies responses to constant experimental conditions, further consideration into understanding diurnally alternating temperatures and varying moisture pulse effects on recruitment processes is also required. Additionally, while the experimental seed batches were closely monitored during the storage period prior to experimentation in our study, we suggest that further studies are required to test the effects of prolonged and varying storage conditions on seed and seedling performance. It has been noted that substandard storage conditions could lead to unfavorable seed aging, which would impact on seed and seedling performance after storage (Finch‐Savage & Bassel, 2016; Long et al., 2014). There were challenges associated with the nestedness of the data that occurred due to treatment combinations recording zero values (e.g., at 35–40°C under water stress for dormant florets; Figure 3) for germination and/or emergence responses. This situation decreased the number of treatment combinations for interactions and limited the comparisons between species, even when dormancy was alleviated. Despite this limitation, our data provide a strong basis that increasing temperatures and water stress on their own are driving significant seedling losses during germination and emergence. Therefore, understanding the interactions of hydrothermal effects across a narrower range of temperature and water stress combinations (Bradford, 2002; Onofri et al., 2018) is a logical next step in mitigating recruitment deficits under climate change. These approaches require further research to maximize dormancy break, which for many arid‐zone grasses can be complex (Adkins et al., 2002; Erickson et al., 2017).

In spite of these possible limitations, it is reasonable to suggest that many plant species in arid ecosystems are already persisting on the extremes of their tolerance envelopes. As warming and drying of these landscapes continue with increased intensity and pace (Allen et al., 2018; Reynolds et al., 2007), there is an urgent need to prioritize mitigation strategies before net losses of vegetation cover accelerate beyond anthropogenic control. Seed sourcing informed by shifting climate envelopes to factor in future climates (Breed et al., 2018; Prober et al., 2015), managed relocation (Commander et al., 2018; Maschinski & Albrecht, 2017), and even landscape modification to enhance the influence of microclimate buffering (Hardegree et al., 2016; Miller et al., 2017) are just some of the practical solutions being considered as mechanisms to improve species resilience into the future. Taken together, these trends point toward increasingly more challenging restoration environments, and the prediction of greater recruitment losses, as a result of temperature and moisture deficits.

The knowledge generated from this study also demonstrates the process‐based challenges facing arid‐zone conservation efforts, particularly for species that are known to have specialized germination niche requirements (Barga et al., 2017; Cochrane, 2020; Morgan & Ebsary, 2020). While natural filtering processes across the lifespan of a plant include a wider range of biotic and abiotic factors, this study highlights the importance of critical germination, emergence, and survival envelopes under temperature and water stress, which are often overlooked by conceptual and quantitative models. It is expected that significant recruitment losses are already occurring in arid ecosystems (Ooi et al., 2009), especially where soil temperatures rapidly increase following rainfall. If temperature rises continue to occur, there may be irreversible changes to arid ecosystems, especially where stress tolerances for germination, emergence, and survival are exceeded. This highlights a significant knowledge gap in general for arid ecosystems. To better understand resilience of arid ecosystems to climate change‐associated pressures, further studies need to address species responses at both population and community levels. The low germination, emergence, and survival are significant challenges for restoration and suggest seed dormancy, temperature, and water stress to be major factors limiting recruitment success. The implications for low germination and survival are an increased risk in restoration failure and further demonstrate the need to increase our understanding in the factors driving/limiting establishment in order to direct targeted intervention strategies for restoration initiatives. From an intervention perspective and to counter‐recruitment losses in disturbed ecosystems, active intervention strategies need to investigate technologies that support plant establishment as a whole, for example, by manipulating seeds prior to seeding to improve germination speed and vigor (Erickson et al., 2017; Gornish et al., 2019), or by manipulating the substrate to help emerging seedlings survive climatically challenging environments (Bateman et al., 2018; Masarei et al., 2020; Muñoz‐Rojas, Erickson, Dixon, et al., 2016; Muñoz‐Rojas, Erickson, Martini, et al., 2016). If all of these measures fail, plant recovery in degraded arid ecosystems may result in less frequent recruitment and increased localized seed bank extinction—ultimately resulting in range shifts and localized extirpation for native species in future climates.

## AUTHOR CONTRIBUTION

**Wolfgang Lewandrowski:** Conceptualization (lead); Data curation (lead); Formal analysis (lead); Investigation (lead); Methodology (lead); Resources (equal); Writing‐original draft (lead); Writing‐review & editing (equal). **Jason Stevens:** Conceptualization (equal); Formal analysis (supporting); Investigation (equal); Methodology (equal); Resources (equal); Supervision (lead); Writing‐review & editing (equal). **Bruce L. Webber:** Conceptualization (equal); Formal analysis (equal); Investigation (equal); Methodology (equal); Resources (equal); Supervision (equal); Writing‐review & editing (equal). **Emma Dalziell:** Data curation (equal); Investigation (equal); Writing‐review & editing (equal). **Melinda Trudgen:** Data curation (equal); Investigation (equal); Writing‐review & editing (equal). **Amber Bateman:** Data curation (equal); Investigation (equal); Writing‐review & editing (equal). **Todd Erickson** **:** Conceptualization (equal); Investigation (equal); Methodology (equal); Resources (lead); Supervision (equal); Writing‐review & editing (equal).

## Supporting information

Supplementary Material

## Data Availability

All data in this manuscript are archived in the Dryad Digital Repository https://doi.org/10.5061/dryad.573n5tb79 (Lewandrowski et al., 2021).
