# Supplementary material for: Global change impacts on arid zone ecosystems: Seedling establishment processes are threatened by temperature and water stress
Source: Ecol Evol. 2021 May 11;11(12):8071–84. doi: 10.1002/ece3.7638 (PMC8216921; doi:10.1002/ece3.7638)
Supplement: Supplementary file 1 — Supplementary Material [file ECE3-11-8071-s001.docx]

Global change impacts on arid zone ecosystems: seedling establishment processes are threatened by temperature and water stress

Wolfgang Lewandrowski^*1,2^, Jason C. Stevens^1,2^, Bruce L. Webber^2,3,4^, Emma Dalziell^1,2^, Melinda S. Trudgen^2,3^, Amber M. Bateman^1,2^ and Todd E. Erickson^1,2^

**Author affiliations:**

^1^Kings Park Science, Department of Biodiversity, Conservation and Attractions, Kings Park, Western Australia 6005;

^2^School of Biological Sciences, The University of Western Australia, Crawley, Western Australia 6009;

^3^CSIRO Health and Biosecurity, 147 Underwood Ave, Floreat, Western Australia 6014

^4^Western Australian Biodiversity Science Institute, 133 St Georges Terrace, Perth, Western Australia 6000

*Supporting Information:*

**Supporting Information S1** Viability of intact florets and cleaned seeds before (laboratory controls) and after the climate chamber experiment (post chamber)**.**

**Supporting Information S2** Summary statistics from GLMMs for seed germination, seedling emergence and seedling survival from the climate chamber study, and from viability decline after the climate chamber study.

**Supporting Information S3** Seed germination, emergence and survival responses from sown intact florets and cleaned seeds from different constant temperature and water stress treatments.

**Supporting Information S4** Seedling emergence speed at different constant temperature and water stress treatments

**Supporting Information S1** Viability of intact florets and cleaned seeds before (laboratory controls) and after the climate chamber experiment (post chamber)**.**

**Figure S1.** Viability testing of intact florets and cleaned seeds before (laboratory controls) and after the climate chamber experiment (post chamber) showing dead, dormant/ alive, germinated and viable proportions. In the laboratory controls, intact florets and cleaned seeds (both *n* = 25 per replicate) were germinated across constant temperatures (25-40°C), followed by a viability assessment (cut-test). The post chamber germination trial was conducted under conditions considered optimal (30°C; 12 h alternating light/dark conditions) after intact florets and cleaned seeds (both *n* = 75 per replicate) were exposed to constant temperatures, followed by a cut-test. Viability is defined as the number of germinated and ungerminated, viable intact florets or cleaned seeds. Error bars indicate standard errors of the mean, *n* =4.

**Supporting Information S2** Summary statistics from GLMMs for seed germination, seedling emergence and seedling survival from the climate chamber study, and from viability decline after the climate chamber study.

**Table S2.1.** Summary statistics from GLMMs conducted on germination, emergence and survival responses in six *Triodia* species evaluating the effects of dormancy state (intact florets versus cleaned seeds), temperature (25-40°C) and water stress (well-watered versus water-limited) conditions. The intercept for the regression was determined as the dormant floret, under well-watered conditions at 25°C for all species

|  | **Germination** | | | | **Emergence** | | | | **Survival** | | | |
| --- | --- | --- | --- | --- | --- | --- | --- | --- | --- | --- | --- | --- |
| *Predictors* | *Log-Odds*  *95%-CI* | *Probabilities*  *95%-CI* | *z-value* | *p* | *Log-Odds*  *95%-CI* | *Probabilities*  *95%-CI* | *z-value* | *p* | *Log-Odds*  *95%-CI* | *Probabilities*  *95%-CI* | *z-value* | *p* |
| Species [*Triodia basedowii*] | -4.19 (-4.75 – -3.63) | 0.01 (0.01 – 0.03) | -14.67 | **<0.001** | 0.90 (0.40 – 1.41) | 0.71 (0.60 – 0.80) | 3.51 | **<0.001** | 2.11 (0.45 – 3.76) | 0.89 (0.61 – 0.98) | 2.49 | **0.013** |
| Species [*Triodia epactia*] | -3.37 (-3.90 – -2.84) | 0.03 (0.02 – 0.06) | -12.38 | **<0.001** | 0.65 (0.22 – 1.09) | 0.66 (0.55 – 0.75) | 2.96 | **0.003** | 2.47 (0.98 – 3.96) | 0.92 (0.73 – 0.98) | 3.24 | **0.001** |
| Species [*Triodia lanigera*] | -5.09 (-5.69 – -4.49) | 0.01 (0.00 – 0.01) | -16.72 | **<0.001** | -0.02 (-0.60 – 0.55) | 0.49 (0.35 – 0.64) | -0.08 | 0.934 | 1.55 (-0.38 – 3.49) | 0.83 (0.41 – 0.97) | 1.58 | 0.115 |
| Species [*Triodia pungens*] | -3.82 (-4.36 – -3.28) | 0.02 (0.01 – 0.04) | -13.94 | **<0.001** | 0.33 (-0.11 – 0.77) | 0.58 (0.47 – 0.68) | 1.45 | 0.146 | 1.05 (-0.45 – 2.55) | 0.74 (0.39 – 0.93) | 1.37 | 0.170 |
| Species [*Triodia vanleeuwenii*] | -5.14 (-5.74 – -4.53) | 0.01 (0.00 – 0.01) | -16.69 | **<0.001** | 0.75 (0.19 – 1.32) | 0.68 (0.55 – 0.79) | 2.62 | **0.009** | 1.19 (-0.65 – 3.03) | 0.77 (0.34 – 0.95) | 1.27 | 0.205 |
| Species [*Triodia wiseana*] | -3.38 (-3.91 – -2.85) | 0.03 (0.02 – 0.05) | -12.47 | **<0.001** | 0.64 (0.22 – 1.06) | 0.66 (0.55 – 0.74) | 2.98 | **0.003** | 0.95 (-0.58 – 2.48) | 0.72 (0.36 – 0.92) | 1.22 | 0.223 |
| Dormancy state [Seed] | 2.84 (2.48 – 3.20) | 0.94 (0.92 – 0.96) | 15.49 | **<0.001** | -1.13 (-1.48 – -0.78) | 0.24 (0.19 – 0.31) | -6.36 | **<0.001** | -0.76 (-1.84 – 0.32) | 0.32 (0.14 – 0.58) | -1.39 | 0.166 |
| Water Stress [Water-limited] | -1.89 (-2.23 – -1.55) | 0.13 (0.10 – 0.17) | -10.89 | **<0.001** | -0.30 (-0.60 – -0.00) | 0.43 (0.35 – 0.50) | -1.98 | **0.048** | 1.61 (0.53 – 2.70) | 0.83 (0.63 – 0.94) | 2.91 | **0.004** |
| Temperature [30°C] | 0.04 (-0.42 – 0.50) | 0.51 (0.40 – 0.62) | 0.17 | 0.867 | -0.00 (-0.36 – 0.35) | 0.50 (0.41 – 0.59) | -0.03 | 0.980 | -0.03 (-1.32 – 1.27) | 0.49 (0.21 – 0.78) | -0.04 | 0.966 |
| Temperature [35°C] | -0.19 (-0.66 – 0.28) | 0.45 (0.34 – 0.57) | -0.80 | 0.425 | -0.26 (-0.62 – 0.10) | 0.44 (0.35 – 0.53) | -1.41 | 0.158 | -2.06 (-3.39 – -0.74) | 0.11 (0.03 – 0.32) | -3.06 | **0.002** |
| Temperature [40°C] | -0.97 (-1.46 – -0.48) | 0.27 (0.19 – 0.38) | -3.89 | **<0.001** | -1.82 (-2.27 – -1.37) | 0.14 (0.09 – 0.20) | -7.90 | **<0.001** | -4.28 (-6.14 – -2.42) | 0.01 (0.00 – 0.08) | -4.52 | **<0.001** |
| Observations | 384 | | | | 282 | | | | 229 | | | |
| Marginal R^2^ / Conditional R^2^ | 0.484 / 0.558 | | | | 0.204 / 0.208 | | | | 0.334 / 0.631 | | | |

**Table S2.2.** Summary statistics from GLMMs on viability responses after intact florets and cleaned seeds were exposed to well-watered conditions (control) or water-limited conditions at 25-40^o^C temperatures in the climate chamber experiment for six *Triodia* species.

|  | **Viability** | | | |
| --- | --- | --- | --- | --- |
| *Predictors* | *Log-Odds*  *95%-CI* | *Probabilities*  *95%-CI* | *z-value* | *p* |
| Species [*Triodia basedowii*] | 4.44 (3.96 – 4.91) | 0.99 (0.98 – 0.99) | 18.33 | **<0.001** |
| Species [*Triodia epactia*] | 3.92 (3.45 – 4.39) | 0.98 (0.97 – 0.99) | 16.35 | **<0.001** |
| Species [*Triodia lanigera*] | 4.08 (3.61 – 4.55) | 0.98 (0.97 – 0.99) | 17.02 | **<0.001** |
| Species [*Triodia pungens*] | 3.44 (2.97 – 3.90) | 0.97 (0.95 – 0.98) | 14.59 | **<0.001** |
| Species [*Triodia vanleeuwenii*] | 3.91 (3.44 – 4.38) | 0.98 (0.97 – 0.99) | 16.32 | **<0.001** |
| Species [*Triodia wiseana*] | 3.47 (3.00 – 3.93) | 0.97 (0.95 – 0.98) | 14.62 | **<0.001** |
| Dormancy state [Seed] | -0.81 (-1.08 – -0.54) | 0.31 (0.25 – 0.37) | -5.82 | **<0.001** |
| Water Stress [Water-limited] | 0.95 (0.68 – 1.22) | 0.72 (0.66 – 0.77) | 6.84 | **<0.001** |
| Temperature [30°C] | -0.93 (-1.34 – -0.53) | 0.28 (0.21 – 0.37) | -4.53 | **<0.001** |
| Temperature [35°C] | -1.41 (-1.81 – -1.01) | 0.20 (0.14 – 0.27) | -6.92 | **<0.001** |
| Temperature [40°C] | -2.17 (-2.56 – -1.78) | 0.10 (0.07 – 0.14) | -10.78 | **<0.001** |
| Observations | 384 | | | |
| Marginal R^2^ / Conditional R^2^ | 0.218 / 0.265 | | | |

**Supporting Information S3** Seed germination, emergence and survival responses from sown intact florets and cleaned seeds from different constant temperature and water stress treatments.

**Figure S3.1** Germination, emergence and survival from the total number of sown intact florets (*n* = 215) from six *Triodia* species at -0.15 MPa (white panels) and -0.35 MPa (grey panels) soil water potentials incubated at constant 25, 30, 35 and 40°C temperatures. Error bars indicate one standard error of the mean, *n* = 4.

**Figure S3.2** Germination, emergence and survival from the total number of sown cleaned seeds (*n* =215) from six *Triodia* species at -0.15 MPa (white panels) and -0.35 MPa (grey panels) soil water potentials incubated at constant 25, 30, 35 and 40°C temperatures. The error bars indicate one standard error of the mean, *n* = 4).

**Supporting Information S4** Seedling emergence speed at different constant temperature and water stress treatments

*Seedling emergence speed*

Cumulative seedling emergence responses from intact florets and cleaned seeds were plotted against time and a three-parameter log-logistic function fitted to determine time to 50 % emergence across experimental treatments. The three-parameter log-logistic function was chosen as it provided the best fit as described by Ritz and Steibig (2005), based on log-likelihood and AIC-index when compared to other non-linear functions. The three-parameter log-logistic function is defined as,

$$F\left( x,b,d,e \right)=\frac{d}{1+\exp\left[ b\left\{ \log\left( x \right)-\log\left( e \right) \right\} \right]}$$

where (*d*) is the upper limit or maximum emergence, (*b*) the *slope* of the curve (*F*) and time (*x),* and (*e*) the 50% point, which is equal at the median response of the curve (= t_50_) (Ritz and Steibig 2005).

**Table S4.** Seedling emergence speed from intact florets (*n* = 215) and cleaned seeds (*n* = 215) at different constant temperature and water stress treatments from six *Triodia* species. Emergence speed is shown as the time to emerge to 50% (T_50_) ± standard error (*n* = 4), modelled using a three-parameter loglogistic function.

| **Intact Florets** | **25^o^C** | | **30^o^C** | | **35^o^C** | | **40^o^C** | |
| --- | --- | --- | --- | --- | --- | --- | --- | --- |
|  | **Well-watered** | **Water-limited** | **Well-watered** | **Water-limited** | **Well-watered** | **Water-limited** | **Well-watered** | **Water-limited** |
| *Triodia basedowii* | 8.6 ± 1.0 | 12.5 ± 0.8 | 6.3 ± 0.5 | 9.5 ± 1.7 | 5.8 ± 0.7 | - | 9.9 ± 1.3 | - |
| *Triodia epactia* | 9.9 ± 0.4 | 10.3 ± 0.6 | 4.9 ± 0.2 | 5.7 ± 0.5 | 5.7 ± 0.4 | 6.3 ± 0.7 | 7.8 ± 0.6 | - |
| *Triodia lanigera* | 6.1 ± 0.4 | - | 4.5 ± 0.3 | - | 6.1 ± 0.4 | - | - | - |
| *Triodia pungens* | 11.2 ± 0.4 | 11.4 ± 6.1 | 7.7 ± 0.5 | 11.2 ± 0.6 | 9.7 ± 0.4 | 11.8 ± 4.3 | 10.4 ± 0.5 | - |
| *Triodia* *vanleeuwenii* | 11.2 ± 0.3 | - | 9.5 ± 1.0 | - | - | - | - | - |
| *Triodia wiseana* | 6.5 ± 0.3 | 8.7 ± 0.3 | 4.6 ± 0.2 | 6.4 ± 0.3 | 6.5 ± 0.3 | 8.1 ± 0.6 | 9.9 ± 0.6 | - |
| **Cleaned Seeds** | **25^o^C** | | **30^o^C** | | **35^o^C** | | **40^o^C** | |
|  | **Well-watered** | **Water-limited** | **Well-watered** | **Water-limited** | **Well-watered** | **Water-limited** | **Well-watered** | **Water-limited** |
| *Triodia basedowii* | 4.1 ± 0.9 | 4.3 ± 0.6 | 3.1 ± 0.6 | 4.1 ± 1.9 | 4.5 ± 0.2 | 4.7 ± 0.7 | 3.1 ± 1.6 | 7.8 ± 1.0 |
| *Triodia epactia* | 4.7 ± 0.4 | 15.7 ± 0.6 | 7.3 ± 0.5 | 7.4 ± 0.3 | 7.8 ± 0.4 | 0.3 ± 0.6 | 10.5 ± 1.2 | 9.9 ± 2.1 |
| *Triodia lanigera* | 5.4 ± 0.2 | 7.5 ± 0.9 | 3.8 ± 0.2 | 7.5 ± 0.6 | 5.1 ± 0.3 | - | - | - |
| *Triodia pungens* | 8.8 ± 0.2 | 11.4 ± 6.1 | 6.0 ± 1.4 | 11.2 ± 0.6 | 7.4 ± 0.2 | 11.8 ± 4.3 | - | - |
| *Triodia* *vanleeuwenii* | 3.7 ± 0.5 | 4.4 ± 1.4 | 3.9 ± 0.4 | 3.3 ± 1.1 | 3.6 ± 0.4 | 3.4 ± 1.8 | 11.4 ± 0.4 | 13.0 ± 2.3 |
| *Triodia wiseana* | 5.6 ± 0.4 | 5.8 ± 0.8 | 3.7 ± 0.4 | 3.7± 0.7 | 5.9 ± 1.1 | 6.0 ± 0.7 | 4.6 ± 1.4 | - |

**Reference**

Ritz, C. & Streibig, J.C. (2005) Bioassay analysis using R. *Journal of Statistical Software,* **12,** 1-22.
